# Impacts of Natural Selection on Evolution of Core and Symbiotically Specialized (*sym*) Genes in the Polytypic Species *Neorhizobium galegae*

**DOI:** 10.3390/ijms242316696

**Published:** 2023-11-24

**Authors:** Evgeny S. Karasev, Sergey L. Hosid, Tatiana S. Aksenova, Olga P. Onishchuk, Oksana N. Kurchak, Nikolay I. Dzyubenko, Evgeny E. Andronov, Nikolay A. Provorov

**Affiliations:** 1 All-Russia Research Institute for Agricultural Microbiology, 196608 St. Petersburg, Russia; evgenii1991.karasev@gmail.com (E.S.K.); sergey.hosid@gmail.com (S.L.H.); tsaksenova@mail.ru (T.S.A.); olony@yandex.ru (O.P.O.); okurchak@yahoo.com (O.N.K.); provorovnik@yandex.ru (N.A.P.); 2 All-Russia Research Institute of Plant Genetic Resources, 190031 St. Petersburg, Russia; nickolai.dzyubenko@gmail.com; 3 Dokuchaev Soil Science Institute, 119017 Moscow, Russia

**Keywords:** *Neorhizobium galegae*, polytypic rhizobia species, evolution of symbiosis, core and *sym* genes, nucleotide polymorphism, natural selection

## Abstract

Nodule bacteria (rhizobia) represent a suitable model to address a range of fundamental genetic problems, including the impacts of natural selection on the evolution of symbiotic microorganisms. Rhizobia possess multipartite genomes in which symbiotically specialized (*sym*) genes differ from core genes in their natural histories. Diversification of *sym* genes is responsible for rhizobia microevolution, which depends on host-induced natural selection. By contrast, diversification of core genes is responsible for rhizobia speciation, which occurs under the impacts of still unknown selective factors. In this paper, we demonstrate that in goat’s rue rhizobia (*Neorhizobium galegae*) populations collected at North Caucasus, representing two host-specific biovars *orientalis* and *officianalis* (N_2_-fixing symbionts of *Galega orientalis* and *G. officinalis*), the evolutionary mechanisms are different for core and *sym* genes. In both *N. galegae* biovars, core genes are more polymorphic than *sym* genes. In bv. *orientalis*, the evolution of core genes occurs under the impacts of driving selection (dN/dS > 1), while the evolution of *sym* genes is close to neutral (dN/dS ≈ 1). In bv. *officinalis*, the evolution of core genes is neutral, while for *sym* genes, it is dependent on purifying selection (dN/dS < 1). A marked phylogenetic congruence of core and *sym* genes revealed using ANI analysis may be due to a low intensity of gene transfer within and between *N. galegae* biovars. Polymorphism in both gene groups and the impacts of driving selection on core gene evolution are more pronounced in bv. *orientalis* than in bv. *officianalis*, reflecting the diversities of their respective host plant species. In bv. *orientalis*, a highly significant (P_0_ < 0.001) positive correlation is revealed between the p-distance and dN/dS values for core genes, while in bv. *officinalis*, this correlation is of low significance (0.05 < P_0_ < 0.10). For *sym* genes, the correlation between p-distance and dN/dS values is negative in bv. *officinalis* but is not revealed in bv. *orientalis*. These data, along with the functional annotation of core genes implemented using Gene Ontology tools, suggest that the evolution of bv. *officinalis* is based mostly on adaptation for in planta niches while in bv. *orientalis*, evolution presumably depends on adaptation for soil niches. New insights into the tradeoff between natural selection and genetic diversity are presented, suggesting that gene nucleotide polymorphism may be extended by driving selection only in ecologically versatile organisms capable of supporting a broad spectrum of gene alleles in their gene pools.

## 1. Introduction

Root nodule bacteria (rhizobia) represent the genetically most thoroughly studied group of symbiotic microorganisms fixing N_2_ in the nodules of legume and some non-legume plants. Being highly effective producers of N compounds for terrestrial ecosystems, these bacteria are of exclusive ecological and agronomic importance. A complicated system of symbiotically specialized (*sym*) genes, including those responsible for nodule development (*nod*) and N_2_ fixation (*nif/fix*), emerged in rhizobia during co-evolution with host plants, which represent the major factors shaping rhizobia natural history [1,2]. 

Based on intensive genetic research, rhizobia are used as a model to address a range of general evolutionary problems, including the role of natural selection in the evolution of beneficial symbioses. At present, this research is focused on factors restricting cheater rhizobia genotypes, which, according to theoretical/computer models, should outcompete beneficial genotypes in plant-associated populations [3,4]. However, it was demonstrated that, in many symbiotic systems, host-specific selective pressures provide a competitive advantage for mutualists, which resist cheater expansion [5,6], and are responsible for the evolution of N_2_-fixing symbiosis towards increased structural complexity and ecological efficiency [7]. 

Up to now, there has been little focus on the forms of natural selection induced by host plants in polymorphic rhizobia populations. Proceeding from computer and experimental simulations, we suggested that individual (Darwinian, frequency-dependent, disruptive) and group (inter-deme, kin) selection may be induced by hosts in associated rhizobia populations at different stages of symbiosis development [8,9]. These selective pressures are based on and responsible for the high *sym* gene diversity in rhizobia populations. However, the tradeoff between genetic polymorphism and natural selection operating in microbial populations remains obscure [10]. As was previously demonstrated, purifying selection usually results in decreased population/gene polymorphism [11], while disruptive and negative frequency-dependent selection results in extended polymorphism [12,13]. In rhizobia populations, balancing selection may be responsible for the coexistence of cheating and beneficial genotypes, which elicits the evolution of symbiosis for an improved efficiency that is compatible with stable polymorphism in symbiont populations [2]. Data on the influence of driving selection on genetic polymorphism are contradictory: it may be increased [14,15,16,17], conserved, or even decreased [18,19,20] by this selection. 

Research of the impacts of natural selection on symbiosis evolution is based on the multipartite genomic structures of rhizobia in which the core parts encoding for housekeeping functions differ in their natural histories from the accessory parts, including *sym* genes [21]. Convenient models for analyzing rhizobia genome dynamics are represented by polytypic species *Neorhizobium galegae*, which are composed of host-specific biovars bv. *orientalis* and bv. *officinalis* (symbionts of *Galega orientalis* and *G. officinalis*), and *Rhizobium leguminosarum*, composed of bv. *viciae* (symbionts of plants from genera *Lathyrus, Lens, Pisum, Vavilovia,* and *Vicia*), bv. *trifolii* (symbionts of genus *Trifolium*), and bv. *phaseoli* (symbionts of *Phaseolus vulgaris*). 

Rhizobia genomes are subjected to multilevel evolution based on the diversification of: (i) *sym* genes resulting in the formation of polytypic species composed of host-specific biovars; and (ii) core genes resulting in the formation of cryptic (twin) species [8]. It was demonstrated that in different rhizobia species, core genes are evolutionarily more conservative than accessory genes [22,23,24], resulting in a closed pangenome structure for core genes but in an open pangenome structure for accessory genes [25,26]. In *R. leguminosarum*, *sym* and core genes differ greatly in nucleotide polymorphism (p-distance) and are phylogenetically non-congruent, suggesting that the evolution of these genes is mostly independent [27]. This independency may result from intensive gene transfer in *R. leguminosarum* populations, wherein chromosomal core genes recombine randomly with *sym* genes located on mobile plasmids [21].

Previously, we demonstrated that the parameters of core and *sym* gene polymorphism are different in *R. leguminosarum* biovars *viciae* and *trifolii* [27]. Cross-inoculation between these biovars is limited and results in non-N_2_-fixing nodules, which are usually underdeveloped and morphologically abnormal [28]. In *R. leguminosarum*, genomes are composed of circular chromosomes and several plasmids, with one of them (pSym) having a size of 200–500 kb harboring the majority of *sym* genes [21]. We suggested that, in *R. leguminosarum*, evolution of *sym* genes is implemented under the impacts of host-induced natural selection [9,27], while the mechanisms for core gene evolution remain obscure. 

In the presented paper, we compare the evolutionary dynamics of *sym* and core genes in goat’s rue rhizobia (*N. galegae*), in which *sym* genes are located on chromids having sizes of over 1600 kb. These circular replicons have a plasmid-type *rep*ABC system combined with core genes that are typically located on bacterial chromosomes, e.g., rRNA and tRNA genes [29]. Previously, we demonstrated [30] that populations of *N. galegae* bv. *orientalis* collected in the North Caucasian region are more polymorphic for *sym* and core genes than *N. galegae* bv. *officinalis* populations. This difference presumably reflects the diversity of the host plant species, which is sufficiently higher in *G. orientalis* than in *G. officinalis*. The difference between the two *N. galegae* biovars for *nif/fix* genes is much more pronounced than for *nod* genes since the host specificity of the compared biovars pertains to N_2_ fixation not nodulation activity [30]. 

The main objective of the presented research was to address the relationship between nucleotide polymorphism and natural selection in the *N. galegae* population composed of biovars *orientalis* and *officinalis*. Their host species, *G. orientalis* and *G. officinalis*, grow in the North Caucasus region with different levels of diversity, which is mirrored in the polymorphism of their microsymbionts. The following methodology was used: (1) according to analysis of the set of complete *N. galegae* genomes from two biovars, a common gene pool was identified, and for each gene of this pool, nucleotide polymorphism (p-distance) and natural selection statistics (dN/dS) were analyzed; (2) correlation analysis was carried out between the p-distance and dN/dS values separately for core and *sym* genes; (3) to search for genes with either similar or contrastingly different polymorphism and selection patterns between the two rhizobia biovars, analysis of polymorphism and selection was carried out in four groups (p-distance or dN/dS values above average in both biovars, below average in both biovars, and two groups with traits differing in opposite directions); and (4) to address the gene functions presumably involved in differential ecological adaptation of each biovar, the similar analysis was carried using Gene Ontology tools in accordance with the same four-group strategy that was used in the analysis of individual core genes. 

In the presented paper, we analyze the impacts of driving/purifying selection on the diversity of core and *sym* genes of *N. galegae*, which allow us to address the molecular and ecological factors of beneficial symbiosis evolution. The obtained results enable us to reveal the mechanisms shaping the evolution of different parts of multipartite rhizobia genomes and to fill some important gaps in our knowledge on the tradeoff between the polymorphism of genes and impacts of natural selection on their evolution. 

## 2. Results

**Figure 1:**
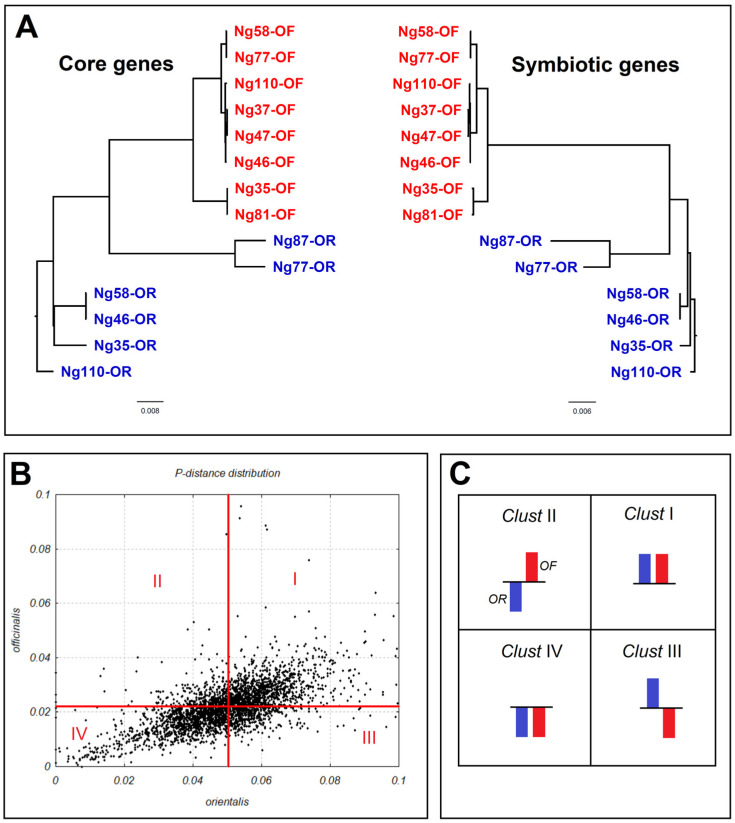
Presentation of the *Neorhizobium galegae* genome and gene datasets studied. (**A**). Phylogenetic congruence of *N. galegae* core (left) and *sym* (right) genes according to average nucleotide identity (ANI) analysis. Strains of bv. *officinalis* (OF) are represented in red, bv. *orientalis* (OR) in blue. (**B**). Distribution of core gene polymorphism (p-distance) in biovars *orientalis* (horizontal axis) and *officinalis* (vertical axis) and division of gene dataset into clusters I–IV. The red lines correspond to the average p-distance value of each biovar. (**C**). The scheme for dividing core genes into four clusters: a column above the line represents a trait value that is above average, below the line represents below average.

In total, 14 complete genomes of *N. galegae* strains (8 bv. *officinalis* and 6 bv. *orientalis*) were studied. In Figure 1A, we present data from the phylogenetic analysis of the average nucleotide identity (ANI) for chromosomal and *sym* genes of this genome set. It should be noted that complete phylogenetic congruence between *sym* and core gene phylogenies reflects parallel processes of microevolution and speciation, probably occurring with lack of horizontal gene transfer. Figure 1B demonstrates different levels of nucleotide polymorphism in individual core genes of bv. *officinalis* and bv. *orientalis*. From this picture it is clear that nucleotide polymorphism is sufficiently higher in bv. *orientalis* (see below for details). Figure 1C shows a scheme of dividing the total core gene pool into four clusters, depending on the similarity/difference in the levels of nucleotide polymorphism of genes in the two *N. galegae* biovars. This scheme was used further for analysis of nucleotide polymorphism and dN/dS in the Gene Ontology group clustering.

### 2.1. Gene Polymorphism and Natural Selection

We demonstrated that nucleotide polymorphism in polytypic species *N. galegae* depends on driving/purifying (dN/dS-measured) natural selection, which operates in a biovar-specific manner in core and *sym* genes (Table 1). The maximal impacts of driving selection (dN/dS > 1) were revealed in the high-polymorphic core genes of bv. *orientalis*, while the minimal impacts were revealed in the low-polymorphic *sym* genes of bv. *officinalis*. 

Analysis of correlations between nucleotide polymorphism (p-distance) and driving/purifying selection (dN/dS) impacts suggested (Table 2) that this selection implements different roles in the evolution of core and *sym* genes, which depends on the *N. galegae* biovar. For core genes, driving selection may result in a marked increase in polymorphism of bv. *orientalis* (indicated by a highly significant positive correlation between p-distance and dN/dS), but this increase was much less evident in bv. *officinalis* (indicated by a significantly lower although positive correlation between p-distance and dN/dS). Figure S1 in the Supplementary Materials demonstrates the corresponding differences in regression between dN/dS and p-distance values in the bv. *orientalis* and bv. *officinalis* core gene pools. 

For *sym* genes, natural selection does not influence polymorphism in bv. *orientalis* (no correlation between p-distance and dN/dS values) but results in a decreased polymorphism in bv. *officinalis* (negative correlation between these values) (Table 2).

Importantly, the frequency of polymorphic *sym* genes in the total gene pools is higher in bv. *orientalis* than in bv. *officinalis*: 38.5 ± 7.8% and 7.7 ± 4.3%, respectively (t_St_ = 3.46; P_0_ < 0.01). For core genes, these frequencies do not differ: 74.6 ± 0.70% and 75.9 ± 0.82%, respectively. 

### 2.2. Gene Ontology Analysis

To address the factors responsible for the evolution of rhizobia core genes, we used Gene Ontology tools [31] to provide the functional annotation of genes that vary sufficiently for nucleotide polymorphism and correlate with the impacts of natural selection (Table 1 and Table 2). The set of 782 core genes that are polymorphic in both *N. galegae* biovars was distributed into 76 Gene Ontology Groups (GOGs), contrasting for p-distance or dN/dS, in which deviations of these parameters exceed the standard deviations of the average values of GO enrichment (1.509 for dN/dS and 1.519 for p-distance). These GOGs were assigned into clusters (as shown in Figure 1C) with contrasting p-distance and dN/dS values: (i) higher than average in both biovars, *orientalis* and *officinalis* (Ori+Off+); (ii) below average in bv. *orientalis* but higher in bv. *officinalis* (Ori–Off+); (iii); higher in bv. *orientalis* but below average in bv. *officinalis* (Ori+Off–); and (iv) below average in both biovars (Ori–Off–). For statistical analysis, four clusters were established independently for p-distance (C_pol_-I…C_pol_-IV) and dN/dS (C_sel_-I…C_sel_-IV) values. 

**Figure 2:**
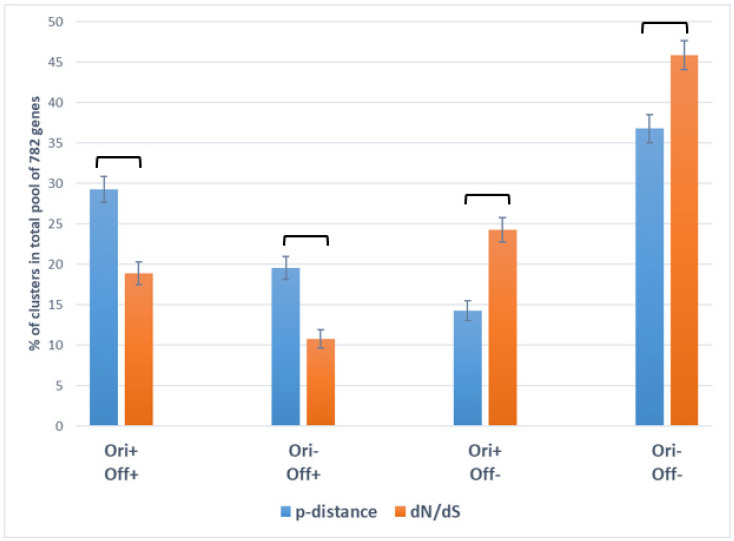
Distribution of 782 polymorphic core genes into clusters (introduced in text) contrasting for p-distance (C_pol_-I…C_pol_-IV are in blue) and dN/dS (C_sel_-I…C_sel_-IV are in orange) values in *Neorhizobium galegae* biovars *orientalis* (Ori) and *officinalis* (Off). The vertical axis shows the representations (in %, with standard errors) of each cluster in the total pool of 782 analyzed genes (data from Table 3 are used, sizes of columns are given in Table S1 in the Supplementary Materials). Columns with significant differences are connected with square brackets.

We demonstrated that clusters C_pol_-IV and C_sel_-IV, in which p-distance and dN/dS values are below average in both *N. galegae* biovars (Ori–Off–), are most numerous, suggesting that purifying selection (dN/dS < 1 in C_sel_-IV cluster) resulting in decreased nucleotide polymorphism (low p-distance in C_pol_-IV cluster) represents an important factor of core gene evolution (Table 3). However, the tradeoff between gene polymorphism and natural selection varies greatly in the analyzed genes: gene frequencies (%) in the total pool of 782 polymorphic genes for the Ori+Off+ and Ori–Off+ clusters are higher for p-distance than for dN/dS (C_pol_-I > C_sel_-I; C_pol_-II > C_sel_-II), while in the Ori+Off– and Ori–Off– clusters, gene frequencies are higher for dN/dS than for p-distance (C_sel_-III > C_pol_-III; C_sel_-IV > C_pol_-IV) (Figure 2).

Analysis of GOG composition enabled us to reveal several regularities in the functional segregation of the core genome concerning its operational (involved in cellular metabolism and development) and informational (involved in template processes) components. As expected, genes that are less variable in both biovars (Ori–Off– clusters with minimal p-distance or dN/dS values for both biovars) are associated with highly conservative template processes. Specifically, genes encoding for translation are revealed in the C_pol_-IV cluster with minimal p-distance while genes for replication, transcription, translation, and DNA repair are in the C_sel_-IV cluster with minimal dN/dS (Tables S2–S4 in the Supplementary Materials). Interestingly, analysis of GOGs identified on the basis of p-distance demonstrated (Table S2 in Supplement) that low-polymorphic genes responsible for metabolism of N-compounds (nucleosides, amino acids) are assigned to C_pol_-III, while low-polymorphic genes for lipid and oligosaccharide metabolism are in C_pol_-II. 

**Figure 3:**
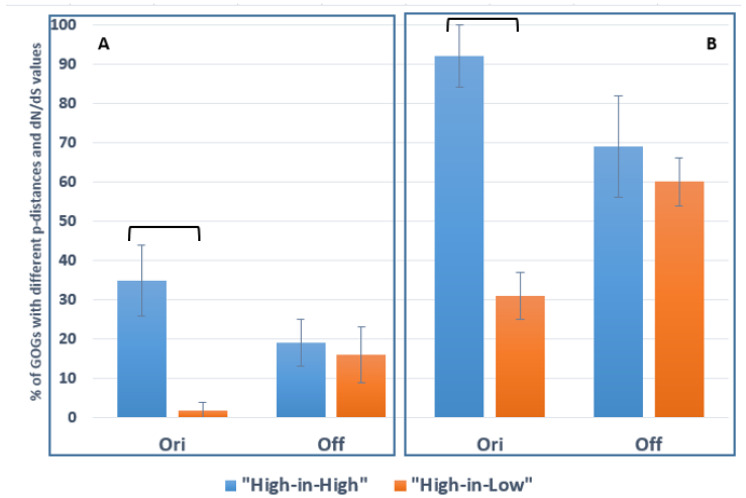
Statistical analysis of clustering of 76 Gene Ontology Groups (GOGs) contrasting for p-distance and dN/dS values in *Neorhizobium galegae* biovars *orientalis* (Ori) and *officinalis* (Off) (data from Table 3 are used, sizes of columns are given in Tables S5 and S6 in the Supplementary Materials). (**A**) Frequencies (in % with standard errors) for GOGs with elevated dN/dS values among GOGs with elevated or decreased p-distance values (“High-in-High” and “High-in-Low” frequencies are presented in blue and orange, respectively). (**B**) The same for GOGs with elevated p-distance values among GOGs with elevated or decreased dN/dS values. Significant (P_0_ < 0.01) differences were revealed in the comparisons of “High-in-High” and “High-in-Low” frequencies for bv. *orientalis*, while for bv. *officinalis*, these differences were not significant. Columns with significant differences are connected with square brackets.

In order to address the tradeoff between nucleotide polymorphism and impacts of natural selection on core gene evolution, we analyzed the distributions of GOGs among the clusters identified using p-distance or dN/dS values (Table 3). To statistically assess the coincidence of these distributions, we separately calculated for the two *N. galegae* biovars the frequencies of GOGs with elevated values of dN/dS or p-distance within GOGs with elevated or decreased values of p-distance or dN/dS (addressed as “High-in-High” and “High-in-Low” frequencies, respectively). This calculation demonstrated that, in bv. *orientalis*, “High-in-High” exceeds “High-in-Low” frequencies for p-distance (Figure 3A) and for dN/dS (Figure 3B), suggesting that driving selection is responsible for elevated core gene polymorphism in this biovar. However, in bv. *officinalis*, no difference was revealed between “High-in-High” and “High-in-Low” frequencies, suggesting a negligable influence of driving selection on gene polymorphism in this biovar. 

## 3. Discussion

The aim of our research was to use *N. galegae* species possessing multicomponent genome to compare the evolutionary dynamics of core and symbiotically specialized parts. In order to reveal the impacts of natural selection on rhizobia gene polymorphism, we analyzed a set of *N. galegae* strains originating from the North Caucasus region. In accordance with previously published results [30], we demonstrated that the diversity of nucleotide sequences (measured as p-distance) in *N. galegae* is higher for core genes than for *sym* genes and is biovar-dependent: bv. *orientalis* is more polymorphic than bv. *officinalis* for both gene groups (Table 1). This difference may be due to contrasting levels of diversity in the respective host plant species. Specifically, North Caucasus is the longstanding center of *G. orientalis* origin while colonization of this area by *G. officinalis* is more recent [32]. Previously, we quantified the diversity of two *Galega* species in North Caucasus using AFLP fingerprinting, followed by nucleotide polymorphism analysis for a range of genes, followed by genomic fingerprinting, which confirmed the morphological data and suggested higher *G. orientalis* diversity with respect to *G. officinalis* [33,34]. An important source of genetic diversity in *N. galegae* may be represented by insertion sequences (IS), which are more abandoned in bv. *orientalis* than in bv. *officinalis* [35]. 

In this paper, we demonstrate that in *N. galegae*, core and *sym* genes are phylogenetically congruent (Figure 1A), apparently due to their restricted recombination based on the location of *sym* genes on non-mobile chromids. Nevertheless, some evolutionarily important parameters of diversity differ in these genes. For example, the tradeoff between nucleotide polymorphism and the evolutionary impacts of natural selection depend on the gene group (core or *sym*) and on the *N. galegae* biovar (*orientalis* or *officinalis*) (Table 1). Differential impacts of natural selection on polymorphism of core and *sym* genes are evident in bv. *officinalis* (r values differ significantly) but not in bv. *orientalis* (r values do not differ), suggesting that the adaptive impacts of these genes are biovar-specific (Table 2).

Analysis of the total gene pools (Table 2) as well as Gene Ontology Groups (GOGs) (Table 3, Figure 3) suggested that driving selection (dN/dS > 1) results in increased polymorphism of core genes in bv. *orientalis* but not in bv. *officinalis*. We suggest that, in bv. *orientalis*, maintenance of newly emerged core gene alleles by driving selection may be combined with preservation of preexisting alleles; therefore, genetic polymorphism of this biovar is elevated. However, in bv. *officinalis*, the newly emerged gene alleles possibly substitute for preexisting ones due to a restricted ecological amplitude of this biovar; therefore, gene polymorphism in this biovar is not changed. This difference is in accordance with the contrasting ecological affinities of the compared *N. galegae* biovars [33]. Specifically, in the North Caucasian region, biovar *officinalis* persists under unfavorable conditions occupied by its host, *G. officinalis*, such as wetlands and saline and acid soils, and should survive mostly due to colonization of endosymbiotic niches. However, bv. *orientalis* persists under more favorable habitats occupied by *G. orientalis*, such as moderately moist and non-saline, neutral soils. Therefore, persistence in soil niches dependent on core gene operation may be more prolonged for bv. *orientalis* than for bv. *officinalis*.

In agreement with the contrasting ecological affinities of *Galega* species, a range of differences between their symbionts were revealed: (i) low polymorphic GOGs are affiliated with N metabolism (apparently responsible for symbiotic adaptations) in bv. *officinalis* and with the synthesis of surface polysaccharides (presumable responsible for adaptations to edaphic stresses) in bv. *orientalis* (Tables S2–S4 in the Supplementary Materials); (ii) *sym* genes evolve under purifying selection impacts in bv. *officinalis*, while neutral evolution was revealed for these genes in bv. *orientalis*; (iii) the evolution of core genes occurs mostly under the impacts of driving selection in bv. *orientalis*, while this evolution is neutral in bv. *officinalis* (Table 1, Table 2 and Table 3, Figure 3). These data are in agreement with the suggestion of the critical role of *sym* genes for bv. *officinalis* survival in the “plant–soil” system. 

Our data suggest that different molecular and selective mechanisms are responsible for the evolution of *sym* and core genes in *N. galegae*. Different natural histories of core and *sym* genes were revealed also in *Sinorhizobium meliloti* species, which harbor *sym* genes on mega-plasmids [23,36], *Mesorhizobium* spp. harboring *sym* genes in highly mobile chromosomal islands [26,37,38], and *Bradyrhizobium* spp. harboring these genes in low-mobil chromosomal clusters [39,40]. A detailed comparison of *N. galegae* core and *sym* gene polymorphism is available with another polytypic (composed of host-specific biovars) species, *R. leguminorarum*. Basic differences between these species pertain to their host ranges, which is narrow in *N. galegae* and broad in *R. leguminosarum* (Table 4). 

The striking differences between these polytypic species were revealed by comparing *sym* and core gene polymorphism. Specifically, these gene groups in *N. galegae* are much more similar in their diversity parameters than in *R. leguminosarum*, as was demonstrated by comparing gene phylogenies that coincide completely in *N. galegae* (Figure 1A) but are discordant in *R. leguminosarum* [27]. This difference may be due to a highly restricted recombination of *sym* and core genes in *N. galegae*, while in *R. leguminosarum*, this recombination is relaxed [21].

Different parameters of nucleotide polymorphism may be revealed by comparing two polytypic rhizobia species for gene variations within and between biovars. Within biovars, variation for *sym* genes is higher in *R. leguminosarum* than in *N. galegae*, reflecting the taxonomic diversities of the hosts, which for *R. leguminosarum* represent different genera (bv. *viciae*) or species (bv. *trifolii*), but *N. galegae* biovars are restricted to a single host species. This is why, in *N. galegae*, *sym* genes are less variable than core genes, while in *R. leguminosarum* biovars, *sym* genes are significantly more variable than core genes (Table S8). 

Marked differences between the two polytypic rhizobia species were also revealed by the genetic comparison of strains representing different biovars (Table S9). Specifically, the divergence for *sym* genes is higher in *R. leguminosarum* than in *N. galegae*, clearly correlating with the divergences of the respective host plants. The other reason for this difference may be represented by intensive *sym* gene transfer in *R. leguminosarum*, which is responsible for the incongruence of the gene phylogenies in this species [27]. In both species, inter-biovar differences for *sym* genes are higher than for core genes. These differences are strongest in *R. leguminosarum*, suggesting pronounced disruptive selection in this species resulting from high taxonomic diversity of the host plants.

Collectively, the presented data suggest that gene polymorphism in the compared rhizobia species depends on: (i) the diversity of the hosts; and (ii) the intensity of *sym* gene transfer in rhizobia populations. An attempt to correlate the diversities of *N. galegae sym* genes with the diversity of its hosts was made earlier, but the relevant correlations were restricted to AFLP fingerprints [33]. In *R. leguminosarum*, we demonstrated a close correlation between the diversities of the *nod*A gene involved in Nod factor synthesis by rhizobia and the *Nfr5* gene responsible for perception of Nod factors by hosts [8]. 

Importantly, the molecular mechanisms responsible for core gene polymorphism are less clear than for *sym* gene polymorphism, since the impacts of selective (presumably, edaphic) factors influencing core genes are poorly understood as compared to the impacts of symbiotically induced selective factors. The application of Gene Ontology tools suggested in our paper may represent a useful approach to reveal the functional basis for the adaptive evolution of core genes in rhizobia. 

The other important issue of rhizobia evolutionary genetics pertains to the tradeoff between natural selection and gene polymorphism, which may be increased by driving selective pressures in an ecologically versatile organism (such as *N. galegae* bv. *orientalis*), enabling broad allelic diversity in the analyzed genes. However, in an ecologically restricted organism (such as *N. galegae* bv. *officinalis*), gene polymorphism is not changed or is even decreased under the impacts of driving selection since co-existence of different gene alleles is presumable blocked. Significantly, in the mutualistic legume–rhizobia system, the genotypic partners’ interactions responsible for plant fitness may stabilize gene variation under the impacts of either driving or purifying selection induced by hosts and environment in microbial populations [41]. Extended genetic and bioinformatics analyses are required to address the relationship between the adaptive potentials of different gene groups and the impacts of natural selection on the polymorphism expressed in diverse rhizobia species and in other symbiotic organisms.

## 4. Materials and Methods 

### 4.1. Collection of Strains and DNA Sequencing

During an expedition to the North Caucasus in 2003, a number of soil samples were collected, from which rhizobia strains of bv. *orientalis* and bv. *officinalis* were isolated [30,33,34]. 14 rhizobia strains were isolated from soil samples in a microvegetation experiment using nodules of *Galega orientalis* and *G. officinalis* according to standard protocol [42]. They included strains of bv. *officinalis* (NG_35_off (JANFGK000000000), NG_37_off (VYYB00000000), NG_46_off (JANFGL000000000), NG_47_off (JAMQCN000000000), NG_58_off (JANFGM000000000), NG_77_off (JANFGN000000000), NG_81_off (JANFGO000000000), and NG_110_off (VZUM00000000)) and of bv. *orientalis* (NG_35_ori (JANFGP000000000), NG_46_ori (JANFGQ000000000), NG_58_ori (VZUN00000000), NG_77_ori (JANFGR000000000), NG_87_ori (VZUL00000000), and NG_110_ori (JANEZU000000000)).

Isolates were cultivated at 28 °C and 220 rpm for 48 h in modified yeast mannitol broth (YMB) with 1% sucrose [43]. DNA was obtained by the lysozyme–SDS–phenol–chloroform extraction protocol, with minor modifications [44]. Sequencing of strains NG_37_off and NG_87_ori was performed on a PacBio RS II instrument with P6 in two SMRT cells (Pacific Biosciences of California, Inc., Menlo Park, CA, USA). PacBio sequencing, subsequent error correction analysis, and assembly were performed at the Arizona Genomics Institute (US). Genome assembly for the strains was carried out de novo using HGAP https://github.com/jtchien0925/PacBio_HGAP_assembly (accessed on 19 November 2023). Sequencing of 12 other rhizobia strains, 7 strains of biovar *officinalis* and 5 strains of biovar *orientalis*, was performed on a MiSeq genomic sequencer (Illumina Inc., San Diego, CA, USA) according to the manufacturer’s protocol, using the MiSeq Reagent Kit 600 Cycles (Illumina, USA), at the Genomics Core Facility, Siberian Branch, Russian Academy of Sciences (Institute of Chemical Biology and Fundamental Medicine, Novosibirsk, Russia). Assembly of the sequences was carried out using the CLC Workbench https://digitalinsights.qiagen.com/products-overview/discovery-insights-portfolio/analysis-and-visualization/qiagen-clc-genomics-workbench/ (accessed on 19 November 2023) by mapping on reference genomes NG_37_off and NG_87_ori of each biovar, respectively.

### 4.2. Finding Core Genes by Global Alignment

All genes of each genome were matched with the genes of the other 13 genomes using the global alignment method. For this, BLAT was used http://genome.ucsc.edu/cgi-bin/hgBlat (accessed on 19 November 2023). All paired genes were sorted in descending order of identity. Paired genes with a maximal identity of at least 70% in the DNA sequences were selected. After that, a table of all genes and their presence in each of the 14 strains was generated. Only genes that were found in all 14 strains were selected for the core genome.

For bv. *orientalis*, the estimated number of core genes was approximately 5000, for bv. *officinalis*, it was 4200, while 3900 core genes were common for the two biovars. For analyzing the variability and selection indices, we used 3840 genes from bv. *orientalis* and 2734 genes from bv. *officinalis* (genes are common for the two biovars with non-zero polymorphism and dN/dS).

### 4.3. Symbiotic Genes

For both biovars, we analyzed 16 *nod* genes (encoding for Nod factor synthesis) 8 *nif* genes (for nitrogenase synthesis), and 15 *fix* genes (for electron and energy supply of nitrogenase) (Table S7 in the Supplementary Materials). 

### 4.4. Gene Alignment Using Muscle

The DNA sequences of 14 strain variants of each gene were aligned by MUSCLE Multiple Sequence Comparison by Log-Expectation, https://www.ebi.ac.uk/Tools/msa/muscle/ (accessed on 19 November 2023) using standard coding sequence alignment parameters.

### 4.5. Calculation of Nucleotide Polymorphism (p-Distance)

The DNA polymorphism of each gene was calculated based on the number of nucleotide substitutions for each pair of strains using standard metrics https://www.megasoftware.net/mega1_manual/Distance.html (accessed on 19 November 2023). DNA regions with undetermined sequences (N, non-detected) and gaps were not taken into account. The number of substitutions was normalized by dividing the total length of the compared genes without gaps and undefined nucleotides. The matrices (sized 14 × 14 by the number of strains) of the p-distances of each gene were calculated. The average polymorphism of each gene was calculated using the average p-distance of all elements of the matrix, excluding diagonal elements (distance of a gene with itself is zero). Scripts are available at https://github.com/sergeyhosid/P-distance (accessed on 19 November 2023). 

### 4.6. Calculation of dN/dS Index

Calculation of the dN/dS ratio of nonsynonymous (dN) to synonymous (dS) substitutions was performed according to the Jukes–Cantor (JC) model https://bioinformatics.cvr.ac.uk/calculating-dnds-for-ngs-datasets/ (accessed on 19 November 2023). In the JC model, dN/dS for each codon was calculated separately and compared with the theorized ratio of substitutions. For example, alanine is encoded by three different codons when there are nine possible single substitutions of each codon and, consequently, its theoretical dN/dS ratio is 3/9 or 1/3. The obtained dN/dS value of each gene was normalized by dividing by the number of coding codons of the compared sequences. Then, the matrices (sized 14 × 14 by the number of strains) of the dN/dS indexes of each gene were calculated. The average dN/dS index of each gene was calculated by the average dN/dS index of all elements of the matrix, excluding diagonal elements. Scripts are available at https://github.com/sergeyhosid/dNdS (accessed on 19 November 2023). 

### 4.7. Functional Annotation of Core Genes, Gene Ontology (GO)

We used eggnog-mapper https://github.com/eggnogdb/eggnog-mapper/issues/135 (accessed on 19 November 2023) to annotate newly assembled genomes and assign genes to certain functional groups based on Gene Ontology. A detailed transcript of each group was performed on the AmiGO 2 website [45]. AmiGO 2 is a project to create the next generation of AmiGO, the current official web-based toolkit for searching and browsing the Gene Ontology database. The Gene Ontology Consortium (GOC) provides computable knowledge regarding the functions of genes and gene products.

### 4.8. Determination of the Predominance of Functional Groups of Genes GO (Gene Ontology) and Statistical Significance

The prevalence of certain groups of genes was calculated as the ratio of the actual number of genes to their expected number based on the sample size and total number of genes of the selected group:

P_enrich_ = N_obs_/(S_sep_*(N_sep_/N_genome_))

where P*_enrich_* is the predominance of a given group of genes, N*_obs_* is the number of genes in the sample, S*_sep_* is the number of genes in a given group (GO), N*_sep_* is the sample size, and N*_genome_* is the total number of genes found in Gene Ontology. 

The statistical significance of the predominance of certain groups of genes was obtained using a permutation test that simulated the same value of the size of the group and the total number of genes. The permutation test was performed 10,000 times, which was sufficient to calculate the statistical significance at 95% (*p*-value < 0.05).

## Figures and Tables

**Table 1:** Nucleotide polymorphism (p-distance) and driving/purifying selection impacts (dN/dS) on core and *sym* gene evolution in host-specific *Neorhizobium galegae* biovars.

Genes *	Means ± Standard Errors		
	bv. *orientalis*	bv. *officinalis*	t_St_ (P_0_)
p-distance			
core	0.048 ± 0.001	0.010 ± 0.001	106.4 (<0.001)
*sym*	0.028 ± 0.008	0.005 ± 0.001	2.84 (<0.05)
t_St_ (P_0_)	2.47 (<0.05)	3.57 (<0.01)	-
dN/dS **			
core	1.571 ± 0.050 (D)	1.013 ± 0.026 (N)	9.91 (<0.001)
*sym*	1.009 ± 0.142 (N)	0.272 ± 0.111 (P)	4.09 (<0.001)
t_St_ (P_0_)	3.72 (<0.01)	6.50 (<0.001)	-

* Numbers of studied core genes are 3840 for bv. *orientalis* and 2734 for bv. *officinalis*; number of studied *sym* genes for both biovars is 39 (16 *nod*, 8 *nif*, 15 *fix* genes are listed in Table S7 in the Supplementary Materials). The Student’s t-test (t_St_) was used to assess the probability of the null hypothesis (P_0_) suggesting no difference between core and *sym* gene groups or between *N. galegae* biovars. ** Natural selection is: D—driving (dN > dS), P—purifying (dN < dS); N—no selection (dN ≈ dS; neutral evolution occurs).

**Table 2:** Correlations between nucleotide polymorphism (p-distance) and natural selection (dN/dS) impacts in core and *sym* genes of *Neorhizobium galegae* biovars.

Genes	Pearson correlations (r) *		t_St_ (P_0_)
	bv. orientalis	bv. officinalis	
core	+0.346 (P_0_ < 0.001)	+0.066 (0.05 < P_0_ < 0.10)	12.73 (<0.001)
sym **	+0.078 (P_0_ > 0.10)	–0.991 (0.05 < P_0_ < 0.10)	4.18 (<0.001)
t_St_ (P_0_)	0.99 (>0.10)	50.03 (<0.001)	-

* Probabilities of the null hypothesis suggesting no correlation between p-distance and dN/dS are given in parentheses after r values; t_St_ (P_0_) used to compare the r values is introduced in Table 1. ** Numbers of studied polymorphic core genes are 2864 for biovar *orientalis* and 2076 for biovar *officinalis*; numbers of studied *sym* genes are 15 for bv. *orientalis* and 3 for bv. *officinalis* (only *sym* genes polymorphic in both biovars were analyzed).

**Table 3:** Distribution of 76 Gene Ontology Groups (GOGs) composed of 782 polymorphic *Neorhizobium galegae* core genes into clusters with contrasting p-distance (C_pol_-I … C_pol_-IV) or dN/dS (C_sel_-I … C_sel_-IV) values (clusters are introduced in the text).

Gene Clusters with Contrasting Values of Polymorphism or Natural Selection		Numbers of GOGs in Clusters Contrasting for p-Distance *				
		C_pol_-I (229)	C_pol_-II (112)	C_pol_-III (153)	C_pol_-IV (288)	Total GOGs
The same in clusters for dN/dS *	C_sel_-I (148)	3	0	0	0	3
	C_sel_-II (190)	4	2	0	4	10
	C_sel_-III (85)	5	1	3	0	9
	C_sel_-IV (359)	12	20	4	18	54
	Total GOGs	24	23	7	22	76

* Numbers of genes in each cluster are given in parentheses.

**Table 4:** Comparison of evolutionarily sufficient items in *Neorhizobium galegae* and *Rhizobium leguminosarum*.

Items	*Neorhizobium * galegae *	Rhizobium leguminosarum **
Compared biovars (their hosts)	bv. *orientalis* (*Galega orientalis*), bv. *officinalis* (*G. officinalis*)	bv. *viciae* (*Lathyrus, Lens, Pisum, Vavilovia, Vicia*), bv. *trifolii* (*Trifolium*)
Taxonomic diversity of host plants (host ranges of compared rhizobia species)	Different species of the same plant genus (narrow)	Different plant genera and tribes (broad)
Replicons harboring *sym* genes	Chromids (>1600 kb)	Plasmids (200–500 kb)
Phylogenetic congruence of core and *sym* genes	High or complete	Low or absent
Differences between biovars for nucleotide sequences of *sym* and core genes (Table S9)	Significant; for *sym* genes, polymorphism is more pronounced than for core genes	Highly significant for *sym* genes but absent or less significant for core genes
Variation within biovars (Table S8): for core genes for *sym* genes	highly significant significant but less than for core genes	significant much higher than for core genes

* This research (Table 1, Tables S8 and S9); ** from [27].

## Data Availability

All 14 genome sequences mentioned in this study can be downloaded by their accession number from https://www.ncbi.nlm.nih.gov (accessed on 19 November 2023).

## Supplementary Materials

The supporting information can be downloaded at: https://www.mdpi.com/article/10.3390/ijms242316696/s1.
