# Supplementary material for: Impacts of Natural Selection on Evolution of Core and Symbiotically Specialized (sym) Genes in the Polytypic Species Neorhizobium galegae
Source: Int J Mol Sci. 2023 Nov 24;24(23):16696. doi: 10.3390/ijms242316696 (PMC10706768; doi:10.3390/ijms242316696)
Supplement: Supplementary file 1 [file ijms-24-16696-s001.zip › ijms-2590271-supplementary.pdf]

Supplementary

Figure S1. Regression between polymorphism (p-distance) and selection (log-normalized dN/dS index) of core genes pool of two biovars: *orientalis* and *officinalis*.

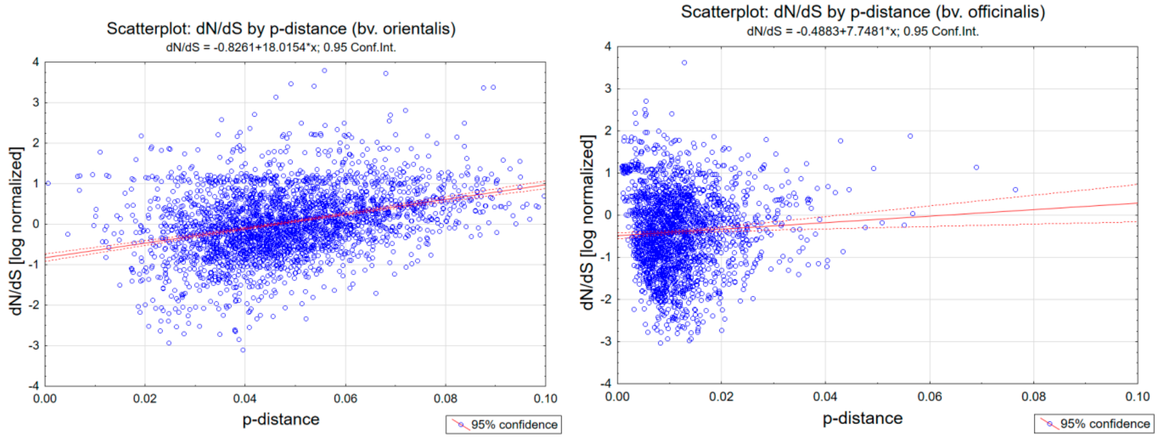

Table S1. Representations (%) of *Neorhizobium galegae* core genes in the clusters identified by the gene nucleotide polymorphism (p-distance) and natural selection (dN/dS) indices (based on analysis of 782 genes which are polymorphic in biovars *orientalis* and *officinalis*).

Indices	Phenotypes of clusters (introduced in the text)			
	Ori+Off+	Ori+Off–	Ori–Off+	Ori–Off–
p-distance	29,3±1,63	14,3±1,25	19,6±1,42	36,8±1,72
dN/dS	18,9±1,40	24,4±1,53	10,8±1,11	45,9±1,78

Table S2. GO (Gene Ontology) enrichment (predominance of functional groups) of biovars *orientalis* and *officinalis* (I), 229 genes, *orientalis* (II), 153 genes and *officinalis* (III), 112 genes. The most enriched 10 functional groups of GO are shown, with a polymorphism (p-distance) value above the average. The last group (IV) *officinalis* and *orientalis*, 288 genes with a polymorphism (p-distance) value below the average. 782 genes were used in the functional analysis.

GO	COG	All genes	Selected	Enrichment	P. val	Description
**(I) *orientalis+officinalis* (p-distance more than average)**		**782**	**229**			
GO:0009396	EFH	6	6	3.41485	0.0005	folic acid-containing compound biosynthetic process
GO:0006040	FGJM	6	6	3.41485	0.0003	amino sugar metabolic process

GO:0042559	EFH	7	6	2.92701	0.0024	pteridine-containing compound biosynthetic process
GO:0006760	EFH	7	6	2.92701	0.0022	folic acid-containing compound metabolic process
GO:0046653	EFH	6	5	2.84571	0.0097	tetrahydrofolate metabolic process
GO:0008170	JS	6	5	2.84571	0.0121	N-methyltransferase activity
GO:0032508	L	5	4	2.73188	0.025	DNA duplex unwinding
GO:0032392	L	5	4	2.73188	0.0274	DNA geometric change
GO:0019200	GQ	5	4	2.73188	0.0263	carbohydrate kinase activity
GO:0017004	OS	5	4	2.73188	0.0254	cytochrome complex assembly
**(II) *orientalis* only (p-distance more than average)**		**782**	**153**			
GO:1901271	IMS	5	4	4.08889	0.0058	lipooligosaccharide biosynthetic process
GO:0033293	CM	5	4	4.08889	0.0062	monocarboxylic acid binding
GO:0009245	IMS	5	4	4.08889	0.0057	lipid A biosynthetic process
GO:0009312	GIMS	7	5	3.65079	0.0053	oligosaccharide biosynthetic process
GO:1901269	IMS	6	4	3.40741	0.014	lipooligosaccharide metabolic process
GO:0046493	IMS	6	4	3.40741	0.0157	lipid A metabolic process
GO:0016791	EFGIPT	6	4	3.40741	0.0153	phosphatase activity
GO:0016311	EFGIPT	6	4	3.40741	0.0144	dephosphorylation
GO:0009311	GIMS	8	5	3.19444	0.0092	oligosaccharide metabolic process
GO:0042578	EFGIKPST	10	5	2.55556	0.0273	phosphoric ester hydrolase activity
**(III) *officinalis* only (p-distance more than average)**		**782**	**112**			
GO:0016840	EF	5	4	5.58571	0.0015	carbon-nitrogen lyase activity
GO:0043023	JMS	5	3	4.18929	0.0248	ribosomal large subunit binding
GO:0009185	FG	5	3	4.18929	0.0251	ribonucleoside diphosphate metabolic process
GO:0009179	FG	5	3	4.18929	0.023	purine ribonucleoside diphosphate metabolic process

GO:0009135	FG	5	3	4.18929	0.0236	purine nucleoside diphosphate metabolic process
GO:0006526	EF	7	4	3.98980	0.0112	arginine biosynthetic process
GO:0006525	EF	7	4	3.98980	0.0111	arginine metabolic process
GO:0031163	EOS	6	3	3.49107	0.0386	metallo-sulfur cluster assembly
GO:0019318	CGM	8	4	3.49107	0.0171	hexose metabolic process
GO:0016226	EOS	6	3	3.49107	0.0406	iron-sulfur cluster assembly
**(IV) *officinalis* and *orientalis* (p-distance less than average)**		**782**	**288**			
GO:0051085	O	5	5	2.71528	0.0066	chaperone cofactor-dependent protein refolding
GO:0051084	O	5	5	2.71528	0.0079	'de novo' posttranslational protein folding
GO:0045259	C	5	5	2.71528	0.0076	proton-transporting ATP synthase complex
GO:0044087	DJKOS	8	8	2.71528	0.0006	regulation of cellular component biogenesis
GO:0043254	KO	5	5	2.71528	0.0069	regulation of protein-containing complex assembly
GO:0042274	JS	15	15	2.71528	0.0001	ribosomal small subunit biogenesis
GO:0034249	JS	5	5	2.71528	0.0071	negative regulation of cellular amide metabolic process
GO:0022625	J	22	22	2.71528	0.0001	cytosolic large ribosomal subunit
GO:0017148	JS	5	5	2.71528	0.0074	negative regulation of translation
GO:0016469	C	5	5	2.71528	0.0092	proton-transporting two-sector ATPase complex

Table S3. GO (Gene Ontology) enrichment (predominance of functional groups) of biovars *orientalis* (646 genes) and *officinalis* (623 genes). The most enriched 10 functional groups of GO with a dN/dS ratio less than one (stabilizing selection) are shown. 782 genes were used in the functional analysis.

**GO**	**All genes**	**Selected**	**Enrchment**	**P. val**	**dN/dS**	**StDev**	**Description**
Orientalis	**782**	**646**					
GO:0098797	17	17	1,21	0,0372	0,403	0,252	plasma membrane protein complex
GO:0097659	18	18	1,21	0,0302	0,291	0,249	nucleic acid-templated transcription
GO:0046031	4	4	1,21	0,0001	0,438	0,287	ADP metabolic process

GO:0032774	18	18	1,21	0,0336	0,291	0,249	RNA biosynthetic process
GO:0009124	16	16	1,21	0,0448	0,38	0,266	nucleoside monophosphate biosynthetic process
GO:0007154	19	19	1,21	0,0282	0,394	0,191	cell communication
GO:0006351	18	18	1,21	0,031	0,291	0,249	transcription, DNA-templated
GO:0006091	31	30	1,17	0,0193	0,437	0,262	generation of precursor metabolites and energy
GO:0016874	44	41	1,13	0,0387	0,426	0,222	Ligase activity
GO:0005515	102	95	1,13	0,0007	0,407	0,216	protein amino acid binding, glycoprotein binding
Officinalis	**782**	**623**					
GO:0072330	18	18	1,26	0,0153	0,574	0,262	monocarboxylic acid biosynthetic process
GO:0071826	31	31	1,26	0,0008	0,366	0,101	ribonucleoprotein complex subunit organization
GO:0070925	31	31	1,26	0,0003	0,369	0,103	organelle assembly
GO:0046364	6	6	1,26	0,0001	0,475	0,164	monosaccharide biosynthetic process
GO:0042886	14	14	1,26	0,0393	0,399	0,283	amide transport
GO:0042274	15	15	1,26	0,0313	0,455	0	ribosomal small subunit biogenesis
GO:0042273	14	14	1,26	0,0428	0,224	0	ribosomal large subunit biogenesis
GO:0042255	30	30	1,26	0,0011	0,34	0,115	ribosome assembly
GO:0033036	16	16	1,26	0,0245	0,349	0,234	macromolecule localization
GO:0022618	30	30	1,26	0,0009	0,34	0,115	ribonucleoprotein complex assembly

Table S4. GO (Gene Ontology) enrichment (predominance of functional groups) of biovars *orientalis* (136 genes) and *officinalis* (159 genes). The most enriched 10 functional groups of GO with dN/dS ratio greater than one (driving selection) are shown. 782 genes participated in the functional analysis.

GO	All genes	Selected	Enrichment	P. val	dN/dS	StDev	Description
Orientalis	**782**	**136**					
GO:0019584	3	3	5.75	0.0053	3.383	2.957	galactonate catabolic process
GO:0019583	3	3	5.75	0.0050	3.383	2.957	galactonate metabolic process
GO:0016855	6	5	4.79	0.0008	1.585	0.359	racemase and epimerase activity, acting on amino acids and derivatives
GO:0047661	5	4	4.6	0.0039	1.595	0.401	L-amino acid racemase activity
GO:0036361	5	4	4.6	0.0032	1.595	0.401	racemase activity, acting on amino acids and derivatives
GO:0046176	4	3	4.31	0.0186	3.383	2.957	aldonic acid catabolic process
GO:0019520	4	3	4.31	0.0195	3.383	2.957	aldonic acid metabolic process
GO:0009103	4	3	4.31	0.0199	1.676	0.408	lipopolysaccharide biosynthetic process

GO:0016854	10	7	4.02	0.0001	1.536	0.376	racemase and epimerase activity
GO:0009074	5	3	3.45	0.0419	3.6	3.606	aromatic amino acid family catabolic process
Officinalis	**782**	**159**					
GO:0019584	3	3	4.92	0.0085	2.073	0.486	galactonate catabolic process
GO:0019583	3	3	4.92	0.0079	2.073	0.486	galactonate metabolic process
GO:0046176	4	3	3.69	0.0287	2.073	0.486	aldonic acid catabolic process
GO:0019520	4	3	3.69	0.0279	2.073	0.486	aldonic acid metabolic process
GO:0006749	4	3	3.69	0.0258	2.694	1.432	glutathione metabolic process
GO:0008168	19	9	2.33	0.0082	1.595	0.57	molecular_function
GO:0008757	17	8	2.31	0.0126	1.564	0.597	S-adenosylmethionine-dependent methyltransferase activity
GO:0019842	13	6	2.27	0.0295	1.924	0.682	vitamin binding
GO:0006400	18	8	2.19	0.0150	1.505	0.394	tRNA modification
GO:0016616	16	7	2.15	0.0264	2.713	1.051	oxidoreductase activity, acting on the CH-OH group of donors, NAD or NADP as acceptor

Table S5. Frequencies (F) of Gene Ontology Groups (GOGs) with high dN/dS values among GOGs with high or low p-distance values

Biovars	p-distances in the compared GOGs				t_{St} (P_0)*
	High-in-High		High-in-Low		
	N (n)	F = n/N	N (n)	F = n/N	
Orientalis	31 (11)	0,35±0,09	45 (1)	0,02±0,02	3,58 (< 0,01)
Officinalis	47 (9)	0,19±0,06	29 (4)	0,16±0,07	0,32 (> 0,10)

N – numbers of GOGs with high or low p-distance values identified in different biovars; n – numbers of GOGs with high dN/dS values (N and n values are calculated from Table 3).

*Students criterion values and probabilities of null-hypothesis are given for F value comparisons.

Table S6. Frequencies (F) of Gene Ontology Groups (GOGs) with high p-distance values among GOGs with high or low dN/dS values

Biovars	dN/dS in the compared GOGs				t_{St} (P_0)*
	High-in-High		High-in-Low		
	N (n)	F = n/N	N (n)	F = n/N	
Orientalis	12 (11)	0,92±0,08	64 (20)	0,31±0,06	6,10 (< 0,01)
Officinalis	13 (9)	0,69±0,13	63 (38)	0,60±0,06	0,33 (> 0,10)

N – numbers of GOGs with high or low dN/dS values identified in different biovars; n – numbers of GOGs with high p-distance values (N and n values are calculated from Table 3).

*Students criterion values and probabilities of null-hypothesis are given for F value comparisons.

Table S7. List of symbiotic *nif, fix* and *nod* genes and their allocation on genomes *Rhizobia Galega* bv. *Officinalis* and *Orientalis*.

Type of symbiotic gene	Gene name	*Officinalis* (st. NG37) Position			Allocation*	Orientalis (st. NG87) Position			Allocation**
NIF	NIFS	2487118	2488587	+	chr	1916477	1917946	+	chr
	NIFH	252902	254401	+	P2	796286	796987	-	P1
	NIFD	254498	256039	+	P2	794896	795792	-	P1
	NIFK	256095	257525	+	P2	793295	794794	-	P1
	NIFE	257544	258890	+	P2	791656	793197	-	P1
	NIFN	259622	259951	+	P2	790170	791600	-	P1
	NIFA	265832	266077	+	P2	782742	783326	-	P1
	NIFB	268251	268487	+	P2	778608	780161	-	P1
FIX	FIXN	501681	502412	+	chr	4500819	4501550	+	chr
	FIXP	503586	505166	+	chr	4502727	4504307	+	chr
	FIXG	505163	505672	+	chr	4504304	4504813	+	chr
	FIXH	505669	507960	+	chr	4504810	4507101	+	chr
	FIXI	507957	508103	+	chr	4507098	4507244	+	chr
	FIXS	508299	508700	+	chr	4507359	4509281	-	chr
	FIXL	2909107	2909727	+	chr	2385220	2385840	+	chr
	FIXK	2911141	2911836	-	chr	2387251	2387943	-	chr
	FIXA	261200	262273	+	P2	787783	788022	+	P1
	FIXB	262266	263573	+	P2	786511	787398	-	P1
	FIXC	263586	263882	+	P2	785364	786437	-	P1
	FIXX	264040	265665	+	P2	784064	785371	-	P1
	FIXU	268785	269984	+	P2	776702	776893	-	P1
NOD	NODT	274696	275265	-	chr	4264116	4264685	-	chr
	NODG_1	985203	986240	+	chr	141852	142889	+	chr
	NODG_2	1780375	1780611	+	chr	1063427	1063663	+	chr
	NODN_1	3726916	3727023	+	chr	3327450	3328232	+	chr
	NODW	1427423	1427794	+	P1	471840	473345	+	chr
	NODO	53984	55684	+	P2	627468	628772	-	P1
	NODN_2	59190	60008	+	P2	712588	713406	+	P1
	NODD2	60099	61160	+	P2	713497	714585	+	P1
	NODE	241845	242123	-	P2	765364	765642	-	P1
	NODF	242595	243158	-	P2	766797	767729	-	P1
	NODD1	246978	247676	+	P2	767978	768568	+	P1
	NODA	247673	248311	+	P2	768565	769209	+	P1
	NODB	248335	249636	+	P2	769233	770534	+	P1
	NODC	249639	250682	+	P2	770537	771580	+	P1
	NODI	250685	251473	+	P2	771748	772371	+	P1
	NODJ	251631	251777	+	P2	772610	772873	+	P1
	NODU	272838	274055	+	P2	1299276	1299437	+	P1

* bv. *Officinalis* (st. NG37): chr is main chromosome (VYYB01000001); P1 (VYYB01000002), P2 (VYYB01000004) are plasmids

** bv. Orientalis (st. NG87): chr is main chromosome (VZUL01000002); P1 (VYYB01000002), P1 (VZUL01000003) is plasmid.

Table S8. Nucleotide polymorphism (p-distance) of *sym* and core genes within biovars of two polytypic rhizobia species

Genes	*Neorhizobium galegae*		*Rhizobium leguminosarum*	
	bv. *orientalis*	bv. *officinalis*	bv. *viciae*	bv. *trifolii*
sym	0.028±0.008	0.005±0.001	0.039±0.005	0.135±0.012
core	0.048±0.001	0.010±0.001	0.019±0.004	0.031±0.007
sym/core	0,58	0,50	2.05	4.40
t_{St} (P_0)	2,49 (< 0.05)	3,57 (< 0.01)	3,33 (< 0.01)	7,43 (< 0.01)

Table S9. Comparison of nucleotide sequences (p-distance) of *sym* and core genes between biovars in two polytypic rhizobia species: *Neorhizobium galegae* (Ng) and *Rhizobium leguminosarum* (Rl)

Genes	Ng	Rl	t_{St} (P_0)
sym	0.080±0.010	0.249±0.012	10,56 (< 0.001)
core	0.071±0.001	0.029±0.009	4,67 (< 0.01)
sym/core	1.13	8.48	-
t_{St} (P_0)	6,36 (< 0.01)	14,66 (< 0.001)	-
